# Internally versus externally trained residents and fellows hired as attendings at a large integrated healthcare system: a 20-year retrospective study

**DOI:** 10.1186/s12960-023-00846-0

**Published:** 2023-07-27

**Authors:** Stephen M. Wertheimer, Jerad A. Harris, J. Craig Collins, Nancy H. Spiegel, Gary W. Chien

**Affiliations:** 1 Wayne State University School of Medicine, Detroit, United States of America; 2 Department of Urology, Kaiser Permanente Los Angeles Medical Center, 4900 Sunset Blvd, 2nd Floor, Los Angeles, CA 90027 United States of America; 3 Department of Surgery, Kaiser Permanente, Southern California, Los Angeles, CA United States of America; 4 Department of Clinical Science, Kaiser Permanente School of Medicine, Pasadena, CA United States of America

**Keywords:** House staff, Hiring your own, Graduate Medical Education, Physician recruitment

## Abstract

**Background:**

There remains a question of whether graduates trained internally are different than those trained elsewhere. We examine the difference between physicians trained within our Graduate Medical Education (GME) programs versus physicians trained elsewhere. Our large integrated healthcare system is unique in addressing this objective due to its large physician labor hiring needs across different specialties of GME graduates.

**Methods:**

A retrospective review was performed from Jan 2000 to August 2020 of Kaiser Permanente Southern California (KPSC) physicians hired: KPSC GME trained versus non-KPSC GME trained. We examined five variables: retention, leadership (current or historical), physician relations cases, member appraisal of physician and provider services survey (MAPPS) scores, and rate of board certification. Chi-square test of proportions was used for comparison, *p* < 0.05 was significant.

**Results:**

From Jan 2000 to August 2020, 2940 residents and fellows graduated from KPSC GME programs, of which 1127 (38%) were hired on at KPSC as full time attendings. Across all five metrics (Retention 82% vs 76% (*p* =  < 0.01), Leadership [current 13% vs 10% (*p* =  < 0.01)or historical 17% vs 14% (*p* = 0.01)], Physician Relations 23% vs 26% (*p* = 0.04), MAPPS 75% vs 69% (*p* =  < 0.01), and Board Certification 81% vs 74% (*p* =  < 0.01)), KPSC outperformed non-KPSC GME-trained physicians to a statistically significant degree.

**Conclusions:**

We have shown that an internally sponsored GME program can represent an opportunity for recruitment of physicians that may have higher retention rates, higher probability of being physician leaders, decreased likelihood of physician relations issues, improved patient satisfaction, and increased rates of board certification.

## Background

The United States is predicted to face a substantial shortage of physicians. Recent estimates project a shortfall of between 41 000 and 105 000 doctors by 2030 [1]. Additionally, national health expenditure (NHE) continues to grow in tandem with the cost of health care per capita. In 2018, NHE grew by 4.6% to $3.6 trillion and accounted for 17.7% of Gross Domestic Product (GDP) [2]. This increase is driven in part by an aging population. Americans older than 65 will make up 21% of the population in 2030, compared with 15% in 2017 [3].

In the setting of rising NHE, there has been increased scrutiny of the expenses required to maintain Graduate Medical Education (GME) programs, manifested by multiple proposals to decrease funding [4, 5]. Between direct and indirect GME payments, largely by Medicare and Medicaid, the mean per-resident payment to hospitals was approximately $139 000 in 2015 [6]. Yet numerous studies have shown the value of GME programs and have demonstrated that eliminating residents results in an increase in the total cost of care [7–10]. Lauer et al. found that surgical GME programs provide significant financial benefit to integrated health care systems, saving between $16 million and $26 million dollars per year [7]. GME programs also provide significant non-monetary value as well, by attracting faculty educators who are invested in training the next generation. Trainees also provide overnight on-call services on behalf of attending physicians; their presence allows for enhanced flexibility, added productivity and greater revenue for a given number of attending staff [7].

As health care costs increase, the population ages, and the physician shortage grows, hospitals will face accelerating challenges in recruiting and retaining talented physicians. Physician recruitment is a time-consuming and costly endeavor that can vary in its rate of success based on hospital location and practice finances. Furthermore, retaining experienced physicians becomes another important area of focus as more doctors report burnout and career changes [11].

For these reasons, the goal of any healthcare system is to hire physicians who possess the particular skills that will equip them to succeed in this challenging environment. The concept of “hire your own” has the obvious benefits of familiarity with the health system and hospital culture, and reduced costs of recruitment. We hypothesize that there may be other quantifiable benefits as well. Can hiring graduates of your own training program improve physician retention, cultivate leadership, and perhaps improve patient satisfaction?

## Methods

This study was approved by our institutional review board. A retrospective review was performed within the Physician Human Resources database of the Kaiser Permanente Southern California (KPSC) Human Resources Department. KPSC is an integrated healthcare system, currently with 15 medical centers serving more than 4.5 million members across Southern California. During the study period from Jan 1, 2000 to August 28, 2020, there were more than 7,000 full time KPSC physicians. KPSC Graduate Medical Education (GME) has been accredited since 1955. It is currently the ACGME Sponsoring Institution for 18 residency and 15 fellowship programs. During our study period, KPSC GME graduated 131–150 residents and fellows annually. Physician names were blinded for the analysis and interpretation of data. All KPSC physicians who were trained either in KPSC residency and/or fellowship from 1/1/2000 to 8/28/2020 were included in the study population. Physicians who trained in KPSC residency followed by any KPSC fellowship were counted as fellowship trained only.

The five dependent variables gathered and analyzed were Retention, Leadership (current or historical), Physician Relations cases, MAPPS (Member Appraisal of Physician and Provider Services) scores, and Board Certification. Active is defined as a physician currently working at least 80% of full time with KPSC. Inactive includes any physician deceased, on leave, or working < 80% of full time. Leadership, current or historical, is defined as any physician who holds or has held a recognized leadership role with salary stipend and/or dedicated administrative time. Physician relations (PR) cases is defined as any record of behavioral and/or performance deficiencies in the KPSC Physician Risk Office. MAPPS score is a standardized patient satisfaction survey that is mailed to randomly selected patients who have had recent interactions with KPSC physicians. All KPSC physicians have an ongoing MAPPS score from their patient encounters that is tallied annually. A score of > 9/10 is recognized as meritorious within the organization and qualifies as “eligible” for this study. Board certification is defined as KPSC physicians who are continuously certified in their primary clinical role (or former clinical role, for executive leaders) by a member Board of the American Board of Medical Specialties.

Statistical analysis was performed using Microsoft Excel. A Chi-squared test of proportions was used for comparison of variables between the two groups. KPSC trained versus non-KP trained physicians were compared. A *p*-value of < 0.05 was considered statistically significant.

## Results

During the study period from Jan 1, 2000 to August 28, 2020, 2940 residents and fellows graduated from KPSC GME programs, of which 1127 (38%) were hired on at KPSC as full time attendings.

Across all five metrics (Retention, Leadership, current or historical, Physician Relations, MAPPS, and Board Certification) for physicians hired by KPSC from January 2000 to August 2020, those who had trained at a KPSC GME program outperformed non-KPSC GME-trained physicians to a statistically significant degree (Table 1).

**Table 1:** KPSC GME-trained versus non-KPSC physicians compared in 5 variables

	Non-KPSC (*n*)	KPSC (*n*)	*p*-value
Retention			
Active	76% (13 071)	82% (925)	< 0.01
Inactive	24% (4198)	18% (202)	
Leadership (current)			
Current leader	10% (1720)	13% (152)	< 0.01
Not current leader	90% (15 549)	87% (975)	
Leadership (historical)			
Former leader	14% (2425)	17% (189)	0.01
Not former leader	86% (14 844)	83% (938)	
Physician Relations			
Has PR case	26% (4515)	23% (263)	0.04
No PR case	74% (12 754)	77% (864)	
MAPPS			
Eligible	69% (11 912)	75% (846)	< 0.01
Not eligible	5% (849)	9% (107)	
Unknown	26% (4508)	15% (174)	
Board certification			
Certified	74% (12 808)	81% (918)	< 0.01
Not certified	26% (4461)	19% (209)	

Retention, measured as active versus inactive KPSC practice status as of August 2020, was significantly greater for KPSC-trained physicians with 82% currently active compared to 76% of non-KPSC trained physicians (Table 1).

Current leadership was measured as holding a recognized leadership position such as chief of service, service line leader, program director, medical director, etc., at KPSC as of August 2020. Significantly more KPSC-trained physicians (14%) currently hold a leadership position compared to non-KPSC trained physicians (10%) (Table 1; *p* < 0.01). For historical leadership, KPSC-trained physicians were more likely to have held a leadership position at any point in their career at Kaiser Permanente compared to non-KPSC trained physicians, 17% vs 14%, respectively (Table 1; *p* = 0.01).

For Physician Relations, 23% of KPSC-trained physicians had a case filed against them during the studied period, compared to 26% of non-KPSC trained physicians (Table 1; *p* = 0.04). MAPPS, a tool for KP members (i.e., patients) to assess physicians or other clinicians, benchmark performance of >  = 9/10 for KPSC trained physicians was 75%, compared to 69% of non-KPSC trained physicians (Table 1; *p* < 0.01).

As of August 27, 2020, 81% of KPSC-trained physicians held an active ABMS board certification, compared to 74% of non-KPSC trained physicians (Table 1; *p* < 0.01).

## Discussion

The concept of “hire your own” has attractive and intuitive benefits. Cultural fit is already understood by both parties, and there is little or no need to orient new hires. Additionally, there is better alignment of expectations between the health care system and physician. In a study by Rivera, in which 120 interviews of “elite professional service firms”, cultural fit is an important factor in hiring and can outweigh concerns about absolute productivity [12]. Yet, to our knowledge, the concept of “fit” and how it impacts other measurable areas of performance has not been studied previously. By conducting a retrospective analysis of KPSC GME hires over the past 20 years, we have demonstrated that physicians who had previously trained either in residency and/or fellowship at KPSC outperformed non-KPSC GME-trained physicians in five key metrics: Retention, Leadership (Current & Historical), Physician Relations, MAPPS, and Board Certification.

We believe KPSC is uniquely suited to address this question for several reasons. As an integrated healthcare system serving over 4.5 million members across 15 medical centers, it has a large physician labor pool requirement for constant, steady physician hires across its comprehensive medical and surgical specialties. This contrasts with most academic GME programs, where full time physician positions are much less likely to be available in proportion to the number of their GME graduates. Many traditional academic program GME graduates are likely to be hired in the community or other healthcare systems. Many, if not most, KPSC graduates will consider working within the KPSC after graduation.

As such, publications like ours are sparse. One study by Kohler et al., investigating physician retention in Michigan, found that 45% of in-state GME graduates practiced within Michigan at some point following graduation [12]. However, this data focused on state-wide retention rather than healthcare system specific retention. Bazemore, in response, argues there may be “imprinting” effects by GME programs on behaviors of trainees that contribute to how and where they practice [13]. To our knowledge, ours is the largest study examining the benefits of a large GME program within a fully integrated health care system that has the ability to hire its own GME graduates into staff physician positions within the medical group (or faculty practice).

There is not currently a standardized GME graduate recruitment process at KPSC. Physician recruitment is heterogenous and can depend on department culture and staffing needs. Over the past 20 years, KPSC GME hires represent just 12.3% of the total physician hires. However, they outperform non-KP GME hires in all five metrics that were analyzed. One of the most significant metrics in terms of overall impact to a healthcare system is retention. Hiring is a costly, time-consuming process, and 82% of physicians hired into KPSC from an internal GME program between 2000 and 2020 are still active, while 76% of non-KPSC GME-trained physician hires are active (202 inactive KPSC GME hires vs 4198 inactive non-KPSC GME hires). This difference in retention could be explained by increased familiarity with institutional culture and understanding of workflows.

Leadership helps inform and establish culture within a health care system. Greater proportions of internal hires who progress to positions of leadership can be beneficial for maintaining institutional knowledge and culture. GME graduate hires will have pre-existing relationships and thus have an assumed advantage in obtaining leadership positions, particularly in positions selected by peers. Proportionally, current and historical leaders have been over-represented by KPSC GME-trained physicians. This could be, at least partly, explained by greater investment in the institution, as well as greater knowledge and understanding of the underlying systems. Leadership has also been shown as critical to a culture of safety within a health care system; safety performance is directly impacted by leadership [14].

Lower rates of Physician Relations cases were seen among KPSC GME-trained physicians. These cases were related to behavioral and/or performance deficiencies that were managed by Physician Risk Officers. Disruptive behaviors or other adverse behavioral issues among physicians can have significant impacts on patient care [15]. While many do not recognize their behaviors, or the stress they are under, organizations still have a responsibility to address any underlying issues. Health care systems that have lower rates of PR cases can expect to expend fewer resources dealing with those deficiencies and to provide better overall patient care.

Patient satisfaction, as recorded by MAPPS surveys, was significantly higher among KPSC GME-trained physicians. Higher patient satisfaction scores are not only important to subjective patient experiences, but are also associated with improved outcomes, including lower 30-day hospital readmission rates [16]. KPSC GME graduates are familiar with the mission and culture of the organization as well as the expectations of health plan members. Higher rates of patient satisfaction could be, in part, due to physicians having prior experience with the patient population and better understanding their needs.

Board certification criteria are determined by each respective specialty and imply that the physician has achieved a minimum level of qualifications and skills. It allows an organization to guarantee a baseline practice standard. Current board certification was found to be significantly higher (81% vs 74%) among KPSC GME-trained physicians compared with non-KPSC trained peers. Higher rates of board certification are commonly associated with higher quality of care [17, 18].

There are some limitations of our study. First, Kaiser Permanente is unique; it is the largest integrated health care system in the United States, and KPSC is one of the largest health care organizations in the state of California. Therefore, our findings may not be fully generalizable to other health care systems or community health care groups. Second, the KP culture is also differentiated by many factors, including a focus on preventive care, and physicians who are salaried rather than paid on a fee-for-service basis. It can be argued that these and other cultural factors attract residents and fellows who preselect for affinity to KPSC, as the organization is well known across the regions it serves. It is also possible that departments may harbor unconscious bias in favoring KPSC GME-trained physicians, improving their chances of leadership roles and/or confronted with less Physician Relations cases, thus resulting in higher retention rates. Furthermore, demographic information such as age, gender and marital status could play a role in retention, as well as exact time of hiring start and stop dates were not examined and may have provided a better baseline comparison of the study groups. Finally, statistical samples are non-random, group sizes are asymmetric, and study variables are non-standard, as previously defined.

However, we strongly believe that this analysis represents an accurate picture of the value that GME-trained hires can bring to their own health care systems, and that the setting in which this study is conducted is unique to address the question asked. We believe these results demonstrate the additional value that internal physician hires can bring to medical groups, integrated systems of care, and their patients. Future research is needed to investigate the potential associated with recruitment cost savings. Further, an in-depth analysis is important to learn why non-KPSC trained physicians, which consists of most of the physicians hired, did not perform as well. Finally, in the era of increasing flexible working schedules, it would be beneficial to study how part-time physicians fare in these metrics in our cohorts.

## Conclusions

In this retrospective study, we demonstrate that internally sponsored GME programs represent an opportunity for teaching hospitals to accrue quality-of-care benefits by recruiting physician graduates from their own programs. These benefits include increased retention, higher probability of ascending to physician leadership, decreased likelihood of Physician Relations issues, improved patient satisfaction, and increased rates of board certification. Recruiting home-grown trainees could save on recruitment costs, integrate new physicians faster into practice, and increase the familiarity of new hires with institutional culture.

## Data Availability

Datasets used and analyzed during current study are available from the corresponding author on reasonable request.

## Abbreviations

GME: Graduate Medical Education
KPSC: Kaiser Permanente Southern California
MAPPS: Member Appraisal of Physician and Provider Services Survey
NHE: National Health Expenditure
GDP: Gross Domestic Product
